# Photoelectron imaging of substituted benzenes in aqueous aerosol droplets

**DOI:** 10.1039/d5cp04376j

**Published:** 2026-02-17

**Authors:** Jonas Heitland, Jong Chan Lee, Grite L. Abma, Simon Schulke, Jan-Henning Friz, Svetlana Tsizin, Bruce L. Yoder, Ruth Signorell

**Affiliations:** a Department of Chemistry and Applied Biosciences, ETH Zürich Vladimir-Prelog Weg 2 8093 Zürich Switzerland jheitland@ethz.ch rsignorell@ethz.ch

## Abstract

Photochemical reactions can be orders of magnitude faster at the surface of water than in bulk solution, possibly due to changes in the stability of electronic ground and excited states. Yet, direct measurements of the interfacial electronic structure of aqueous reactants remain scarce, making it challenging to establish a clear connection between macroscopic photoreactivities and the underlying molecular-level electronic structure. Here, we employ surface-sensitive ultraviolet (UV) photoelectron velocity-map imaging to probe the valence electronic structure of 13 substituted benzenes at the interface of submicrometer-sized aqueous aerosol droplets. The droplet environment induces vertical binding energy (VBE) shifts of several electronvolts relative to the gas phase for aromatic anions, while neutral solutes show more modest gas-to-solution shifts. Increasing the solute concentration may shift the VBEs of some neutral, protic benzene derivatives, possibly due to increased solute–solute interactions such as hydrogen bonding or π-stacking. In contrast, their anionic conjugate bases show no such shift, likely due to electrostatic repulsion, preventing short-range solute–solute interactions. Changes in droplet surface tension and coverage were quantified through concentration-dependent photoelectron yields. The measured data reveal that 300-nm droplets require a 10 000-fold higher concentration of a proxy nonionic surfactant (Triton X-100) than macroscale solutions to achieve an equivalent surface tension. This observation exemplifies the altered surface partitioning behavior in submicron droplets. It underscores the necessity to account for significant solute depletion in the interior of droplets with considerable surface-to-volume ratios. Phenol–water clusters (170 water molecules) and dilute aqueous phenol droplets (50 mM) exhibit matching valence electronic structure, confirming surface selectivity in UV droplet photoelectron imaging and validating cluster studies as models for interfacial solvation.

## Abstract 2

Aromatic molecules at water droplet surfaces: photoelectron imaging quantifies how interfacial hydration reshapes electronic structure and how the vast surface-to-volume ratio of submicron droplets alters surfactant partitioning.

## Graphical abstract

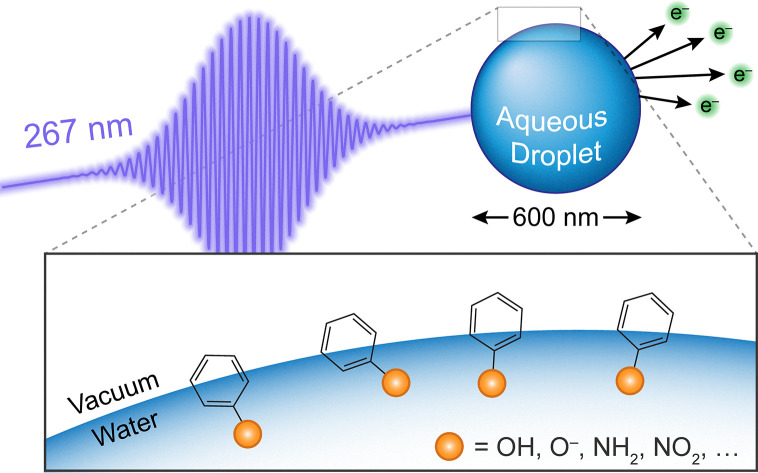

## 1. Introduction

Substituted benzenes represent a major class of atmospheric volatile organic compounds (VOCs), with global emissions exceeding 20 TgC per year from anthropogenic sources including combustion processes, solvent use, and industrial activities.^1–4^ These aromatic compounds are secondary organic aerosol (SOA) precursors and organic constituents of brown carbon (BrC) aerosol particles.^4–9^ Due to their conjugated π-electron systems, these aromatic compounds contribute to the absorption of visible (vis) and near-ultraviolet (UV) solar radiation by BrC aerosol particles, thereby influencing aerosol optical properties and radiative forcing, and affecting tropospheric photochemistry by attenuating actinic flux.^9–11^

The electronic structure and UV-response of substituted benzenes in aqueous droplets are highly relevant to atmospheric photochemistry. Aerosol droplets act as microcompartments, where interfacial reactivity can dominate over bulk reactivity^12,13^ and where photochemical reactions can be modified or accelerated by orders of magnitude relative to macroscale solutions.^7,12,14–25^ This interfacial rate acceleration may result from altered electronic absorption or differences in the stability of the ground and excited states.^7,14,18,20–22,26^ However, direct measurements to elucidate the altered electronic structure and UV-response of solutes at aqueous interfaces remain scarce.^26–30^ This scarcity of direct experimental evidence makes it difficult to establish a clear connection between observed macroscale photoreactivity and the underlying molecular-scale electronic structure.^26^

Photoelectron spectroscopy (PES) on liquid microjets (LJs) provides a straightforward probe of the electronic structure of solutes at the water interface, enabling a comparison with gas-phase ionization energies to directly quantify how electronic structure is modified by the water surface.^26,31–42^ Recently, droplet PES emerged as a complementary method,^26,29,43–47^ offering distinct advantages: Droplets enable the use of photoelectron velocity-map imaging (VMI) and hence angular multiplexing, yielding the full photoelectron angular distribution (PAD) in a single measurement.^29,44,46^ The PAD carries information on orbital character of sample molecules in the gas phase, electron scattering and escape in liquids, and on finite-size effects in aerosols, such as optical cavity resonances.^11,29,44,46^ Furthermore, the PAD enables *in situ* droplet sizing, which is integral to this work.^29^

Droplets are well-suited for probing surface adsorption equilibria – the partitioning between bulk and interface.^26^ Not only do droplets have more time to equilibrate between generation and probing than LJs (seconds *vs.* tens of microseconds), but also – due to the system-size difference – the diffusion timescales in droplets (tens of microseconds) are much shorter than in LJs (tens of milliseconds).‡‡Assuming a 300 nm droplet radius, a tube between atomizer and ADL with a length of 50 cm, an inner diameter of 0.19 in, and a flow rate of 10^−5^ m^3^ s^−1^, a 10 µm LJ radius, a 20 m s^−1^ LJ velocity,^48,49^ a laser–liquid interaction at <1 mm downstream,^49^ and a diffusion constant of phenol in water of 10^−9^ m^2^ s^−1^.^50,51^

Here, we present a systematic investigation of 13 benzene derivatives in submicrometer-sized aqueous droplets using photoelectron VMI spectroscopy. We quantify ionization/detachment energies, total photoelectron yields, and surface coverage – a measure of changes in surface tension – as a function of solute concentration, where possible.

**Fig. 1:**
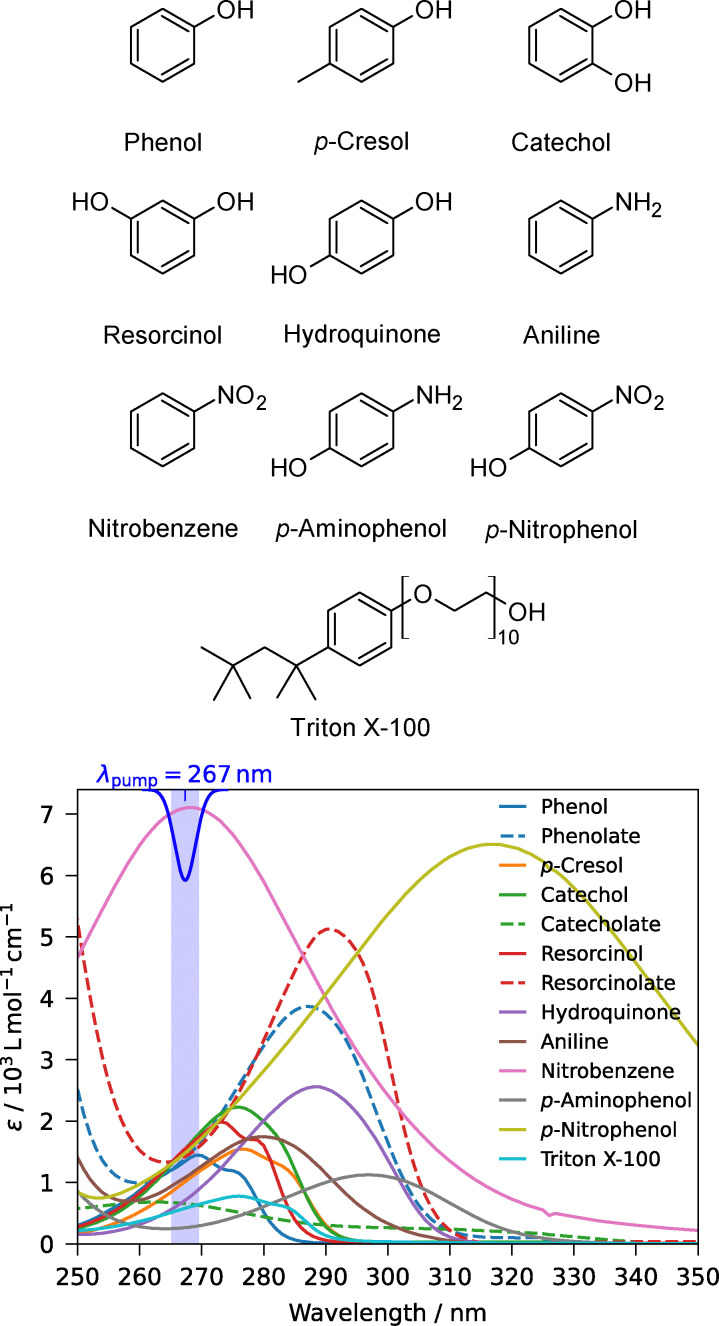
Structures (top) and UV-vis absorption spectra (bottom) of the neutral substituted benzenes (solid lines) and anionic conjugate bases (dashed lines) in aqueous solution recorded using a PerkinElmer LAMBDA 2 UV-vis spectrometer. The blue Gaussian marks the spectral profile of the 267-nm fs pulse used for one-color, two-photon photoelectron spectra, and the shaded region marks its full width at half maximum.

To effectively isolate the weak signal of the dilute solutes from the potentially overwhelming solvent background, we employ resonance-enhanced two-photon ionization/detachment at 267 nm (4.6 eV per photon) with 50-fs or 8-ns pulses. This resonant excitation scheme is crucial for solute selectivity: the photon energy is resonant with the intermediate π → π* singlet excited states of the aromatic solutes (Fig. 1) but lies well below the absorption threshold of water. Furthermore, the total two-photon energy (9.2 eV) is sufficient to ionize the solutes (VIEs ∼7.5–8.5 eV) but remains well below the ionization threshold of liquid water (∼11.3 eV (ref. 35, 36, 52 and 53)), which would require a three-photon process. This energetic selectivity ensures that the photoelectron signal originates almost exclusively from the surface-active solutes.

The aqueous aromatic solutes studied comprise phenol, sodium phenolate, catechol, sodium catecholate, resorcinol, sodium resorcinolate, hydroquinone, *p*-cresol, aniline, nitrobenzene, *p*-aminophenol, *p*-nitrophenol, and the surfactant Triton X-100 (Fig. 1).

Triton X-100 exhibits UV absorption similar to phenol due to its *p*-octylphenoxy moiety. It was included as a well-characterized, nonionic, UV-absorbing surfactant to explore the effects of its vastly increased surface activity (relative to the other benzene derivatives) on the droplet photoelectron signal and to enable quantitative comparison of surface-coverage effects in submicrometer droplets *versus* macroscale solutions.

For all studied benzene derivatives in aqueous solution, the excitation energies and bandwidths corresponding to the singlet excited states populated by absorption of a single photon are presented in the UV-vis absorption spectra in Fig. 1.

## 2. Experimental methods

The following chemicals were purchased and used without further purification: phenol (≥99%, Sigma-Aldrich), *p*-cresol (99%, Sigma-Aldrich), catechol (≥99%, Sigma-Aldrich), resorcinol (99%, Sigma-Aldrich), hydroquinone (99%, Sigma-Aldrich), aniline (99.5%, Thermo Scientific), nitrobenzene (99%, Acros Organics), *p*-aminophenol (≥98%, Sigma-Aldrich), *p*-nitrophenol (≥99%, Sigma-Aldrich), and Triton X-100 (electrophoresis grade, Thermo Scientific). Aqueous solutions were prepared using ultrapure water (HPLC grade, resistivity >18.2 MΩ cm, Sigma-Aldrich). The sodium salts of the deprotonated anionic conjugate bases were prepared by adding sodium hydroxide (≥98%, Sigma-Aldrich) to the aqueous solutions of the corresponding neutral compounds.

**Fig. 2:**
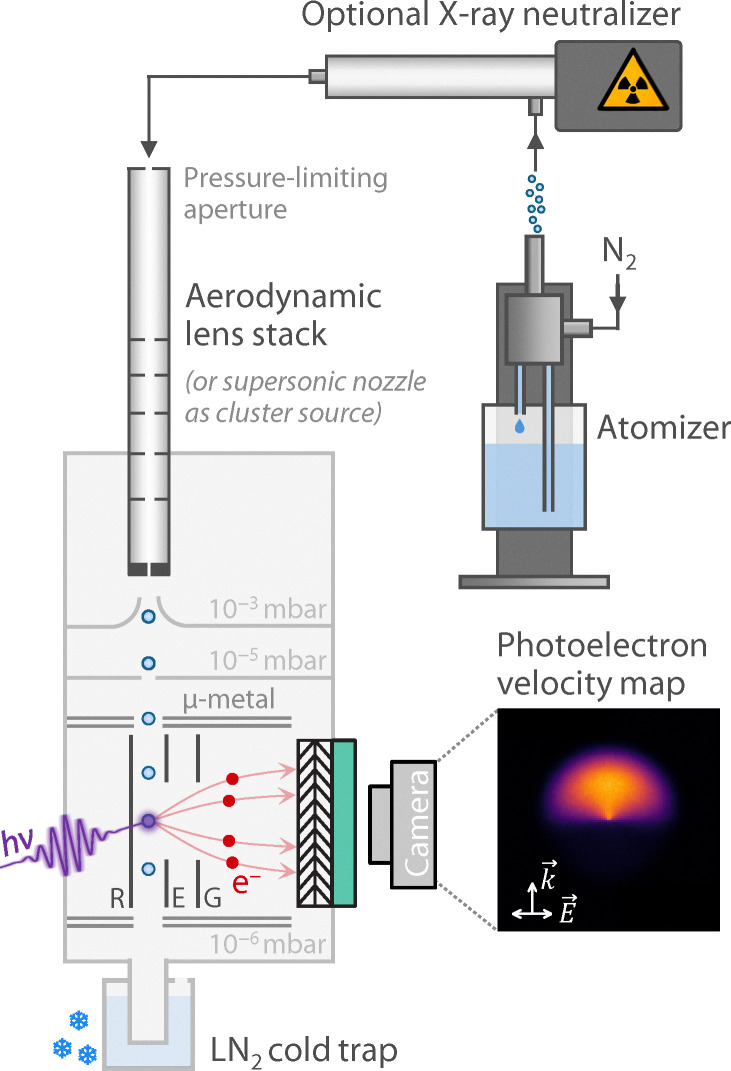
Sketch of the photoelectron VMI spectrometer. Droplets are generated using an atomizer, optionally neutralized, transferred to vacuum and collimated by an ADL, and ionized by fs/ns 267-nm laser pulses. Photoelectrons are velocity-mapped onto a position-sensitive detector (MCP, phosphor screen, camera) using a three-plate electrostatic lens.

Photoelectron images were recorded using a VMI spectrometer, as shown in Fig. 2 and described previously.^43,54^ Aerosol droplets were generated with an atomizer (TSI 3076), optionally charge-neutralized (TSI 3088), and collimated and transferred to vacuum by an aerodynamic lens stack (ADL). The aerosol beam traversed a differential-pumping stage and entered the VMI interaction region, where it crossed a laser beam before impinging on a liquid-nitrogen cold trap to suppress gas-phase background. Droplets were ionized by one-color, two-photon ionization at 267 nm (4.6 eV per photon) using either single femtosecond (fs) or nanosecond (ns) laser pulses. Fs pulses (≤50 fs measured by autocorrelation) were generated by an optical parametric amplifier (Coherent OPerA) pumped by the output (795 nm, ≤40 fs, 1 kHz) of a one-box Ti:sapphire regenerative amplifier (Coherent Astrella); ns pulses (8 ns) were produced from the 4ω of a Q-switched, lamp-pumped Nd:YAG laser (Quantel Ultra 50 Stable) with a repetition rate of 20 Hz. The laser polarization was linear and parallel to the detection plane.

Photoelectrons were velocity-mapped with a three-plate electrostatic lens^55^ onto a position-sensitive gated chevron microchannel plate (MCP)/phosphor detector imaged by a kHz camera. Frames were centroided prior to summing. Images encode electron kinetic energy (eKE) and photoelectron angular distribution (PAD). 3D velocity distributions and eKE spectra were reconstructed from centroided photoelectron images *via* three-point Abel inversion^56,57^ along the laser propagation direction (only cylindrical symmetry axis in droplet VMI^46^), then converted to electron binding energy (eBE) using

1 eBE = 2*hν* − eKE.

Energy calibration was performed with Xe 2 + 1 resonance-enhanced multi-photon ionization (REMPI) at 250 nm. Vertical ionization energies (VIEs) of neutrals and vertical detachment energies (VDEs) of anions are collectively referred to as vertical binding energies (VBEs) and derived from the maxima of the eBE spectra.

The mean droplet radius (∼300 nm) was determined *in situ* from VMI asymmetry and refractive index data as described in ref. 29. After transfer to vacuum, evaporative cooling rapidly supercools these droplets to 240 K within tens of microseconds, with <4% change in radius.^29,45,58,59^

During nebulization, aerosols may acquire electric charges – depending on nebulization method, substance properties, and selected conditions – that can distort photoelectron trajectories and bias the recorded eKEs.^60^ To mitigate this, the aerosols pass through a bipolar diffusion charger ('neutralizer', TSI 3088), exposing them to a bipolar ionic atmosphere generated by a soft X-ray source. Through frequent collisions with charged particles, the aerosols acquire a stationary Boltzmann charge distribution symmetrically centered around zero, rendering the ensemble net neutral. We recorded spectra with and without the neutralizer and observed no changes in the VMIs or extracted VBEs within our experimental uncertainty (±0.1 eV), indicating that droplet charging effects were negligible in our setup. However, passing the aerosols through the neutralizer drastically reduced the signal; therefore, the spectra shown here were recorded without it.

Water–phenol clusters were generated by supersonic expansion of Ar seeded with water and phenol through a pulsed Even–Lavie valve,^61^ and measured with a similar VMI architecture as for aerosols.^54,62^

## 3. Results and discussion

### 3.1. Analysis of the photoelectron images

**Fig. 3:**
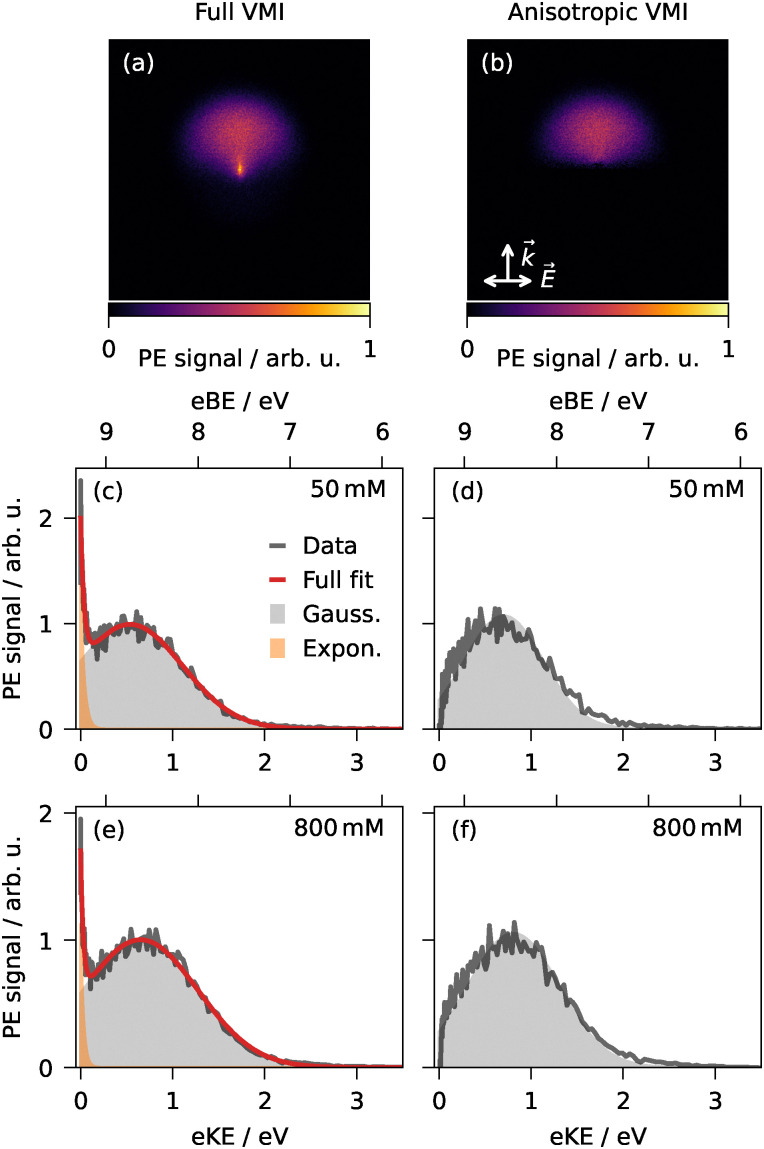
Phenol: Photoelectron spectra and VMIs of phenol in aqueous droplets from single-pulse femtosecond 1 + 1 resonance-enhanced two-photon ionization at 267 nm. Full (a) and corresponding anisotropic VMI (b) from an 800-mM solution. Spectra (gray) with fit (red) as retrieved from the full (c and e) and anisotropic VMIs (d and f) at 50 and 800 mM. A rrows indicate laser propagation (*k⃑*) and polarization (*E⃑*) directions.

**Fig. 4:**
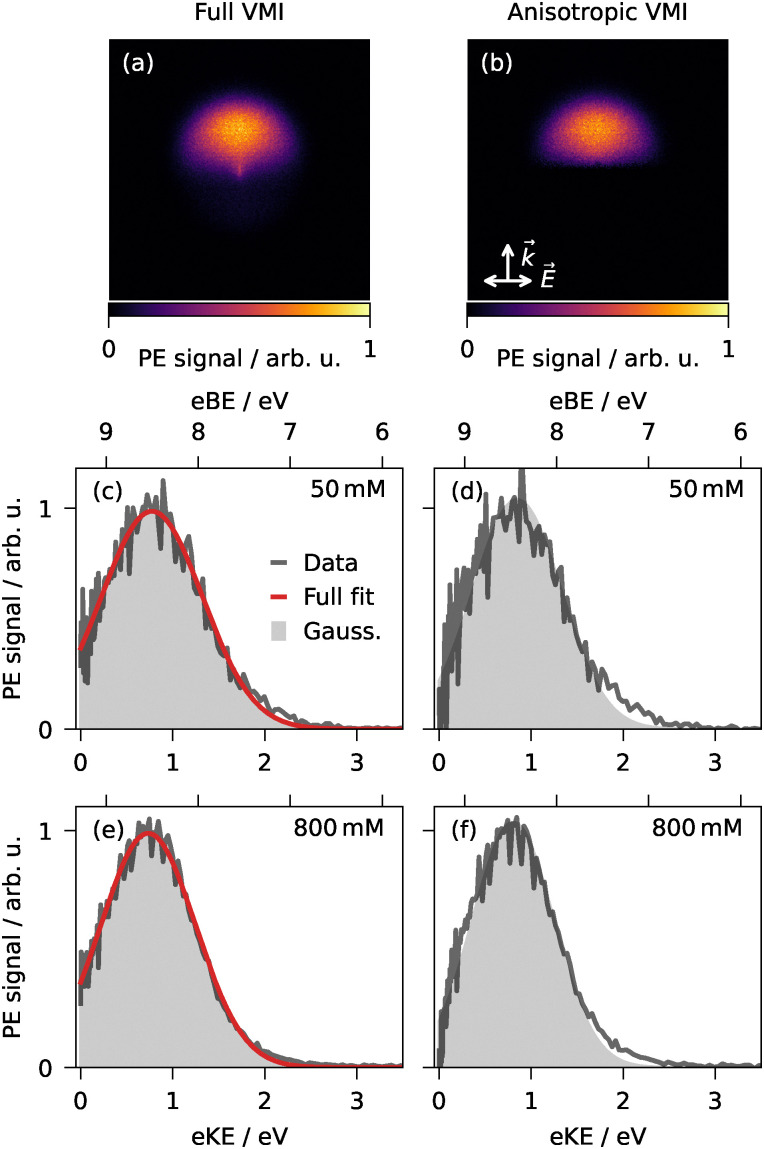
Phenolate: Photoelectron spectra and VMIs of phenolate in aqueous droplets from single-pulse femtosecond 1 + 1 resonance-enhanced two-photon ionization at 267 nm. Full (a) and corresponding anisotropic VMI (b) from an 800-mM solution. Spectra (gray) with fit (red) as retrieved from the full (c and e) and anisotropic VMIs (d and f) at 50 and 800 mM. Arrows indicate laser propagation (*k⃑*) and polarization (*E⃑*) directions.

As observed in our previous work on aqueous phenol droplets,^29^ the VMIs of all substances exhibit a forward–backward asymmetry, revealing that photoelectrons are preferentially (∼80%) emitted in the direction of the laser propagation (*e.g.*, Fig. 3a and 4a). This asymmetry arises from nanofocusing: a local light intensity enhancement in a hot spot at the rear of a droplet, where photoelectrons are then preferentially generated.^11,44,63^ Nanofocusing is characteristic of dielectric particles with sizes comparable to the incident light's wavelength and small absorption cross sections for the incident light.^11,44^ A strong nanofocusing effect, as observed for all substances here, suggests that the droplets' internal light-intensity distribution is governed by the complex refractive index of water rather than the UV-absorbing solutes.^29^

The extent of the forward–backward asymmetry depends on droplet size and refractive index at a given wavelength. Together with the refractive index of water, it was leveraged to determine an average droplet radius of ∼300 nm *in situ* as described in more detail in ref. 29.

The photoelectron spectra were extracted from the VMIs as described in Section 2. Note that the nanofocusing-induced asymmetry of the PAD does not affect the corresponding photoelectron spectra, as these are extracted by integrating the VMI signal over all emission angles as a function of radius (eKE). Representative VMIs and corresponding spectra are presented for phenol and phenolate at two concentrations in Fig. 3 and 4, respectively. The eKE spectra typically exhibit a broad band peaking around 0.5 to 1 eV. In addition to this broad feature, the fs spectra of all neutral species except Triton X-100 show a comparatively strong near-zero kinetic energy (KE) signal, which is not present in any anion or ns spectra. For instance, phenol (Fig. 3a, c and e) displays a strong near-zero-KE signal, while phenolate (Fig. 4a, c and e) does not. The near-zero-KE signal could conceivably arise from contributions from the vapor-phase molecules around the droplets, whose eBEs are higher than those of their aqueous counterparts (see Section 3.2), so their photoelectron signal is expected at correspondingly lower eKEs (eqn (1)).

The aforementioned VMI asymmetry can serve as a diagnostic tool to distinguish the droplet-phase signal from the gas-phase background. Gas-phase contributions to the VMIs are expected to be mainly isotropic as photoemission from randomly oriented gas-phase molecules typically yields photoelectron distributions that exhibit cylindrical symmetry around the laser polarization axis and forward–backward symmetry with respect to the perpendicular laser propagation direction. In contrast, the droplet-phase contribution exhibits a forward–backward asymmetry along the propagation axis due to nanofocusing, as discussed above.^44,46^ To gauge how strongly the isotropic and anisotropic features correlate with each other and to suppress potential isotropic gas-phase contributions, we generated anisotropic VMIs (*e.g.*, Fig. 3b and 4b) by subtracting the front half of the image relative to the laser propagation from the rear half before reconstruction. In the resulting spectra (*e.g.*, Fig. 3c*vs.* d), any near-zero-KE contribution is removed, while the broad main feature remains virtually unaffected, with only minimally shifted peak maxima (<0.2 eV for phenol and even less for dihydroxybenzenes or anions). Essentially, this verifies that the primary broad feature in the spectra originates from the aromatic compounds solvated in the droplet and can be used for further discussion of droplet-phase VBEs.

All eKE spectra were fitted with a Gaussian (gray in Fig. 3 and 4), optionally supplemented by an exponential term (orange in Fig. 3) to model the near-zero-KE feature, where present. The exponential contribution was consistently <6% of the total integrated intensity, corroborating that the near-zero-KE signal can be neglected for further analysis. The centers of the fitted Gaussians were assigned as the apparent two-photon VBEs (eqn (1)) for each compound and are listed in Table 1. The good representation of most spectra by single Gaussians indicates that distortion from in-droplet electron transport and inelastic scattering is minor.^29,36–38^ This observation is consistent with photoemission from surface-active aromatic molecules enriched at the droplet–vacuum interface,^29,36–38,40,42,64^ as previous Monte Carlo electron scattering simulations by our group and others substantiate that photoelectrons generated within a nanometer of the droplet surface experience only minimal energy loss.^29,36–38,65^

**Table 1:** Probed concentration ranges, measured ranges of peak maxima eKE_max_ in the eKE distributions, full widths at half maximum (FWHMs) of the Gaussian fits, apparent two-photon vertical electron binding energies (VBEs), and concentration-induced VBE shifts (ΔVBE) for all substances from the 267-nm two-photon photoelectron spectra of aqueous droplets

Substance	fs/ns	Conc. (mM)	eKE_max_ (eV)	FWHM (eV)	VBE (eV)	ΔVBE (eV)
Phenol	fs	50–800	0.7–0.9	1.1–1.2	8.6–8.4	−0.2
Phenol	ns	10–800	0.8–0.9	∼1.1	8.5–8.4	−0.1
Phenolate	fs	25–1200	0.8	∼1.0	8.5	∼0
*p*-Cresol	ns	5–200	1.0	∼1.3	8.3	∼0
Catechol	fs	50–800	0.9–1.1	1.1	8.4–8.2	−0.2
Catechol	ns	25	1.0	1.2	8.3	n/a
Catecholate	fs	25–400	1.0	∼1.4	8.3	∼0
Catecholate	ns	25	1.0	1.3	8.3	n/a
Resorcinol	fs	50–800	0.6–0.7	0.8–0.9	8.7–8.6	−0.1
Resorcinol	ns	10–100	0.8	1.0	8.5	∼0
Resorcinolate	fs	50–400	0.8	1.1–1.0	8.5	∼0
Hydroquinone	fs	25–200	0.5	∼0.8	8.8	∼0
Hydroquinone	ns	1–200	0.6	∼0.8	8.7	∼0
Aniline	fs	50–200	0.7–0.8	∼1.0	8.6–8.5	−0.1
Aniline	ns	25	0.9	1.4	8.4	n/a
Nitrobenzene	ns	25	0.9	1.2	8.4	n/a
*p*-Aminophenol	fs	25	0.7	1.2	8.6	n/a
*p*-Nitrophenol	fs	25–50	0.5	0.7–0.9	8.8	∼0
*p*-Nitrophenol	ns	25	0.3	0.5	9.0	n/a
Triton X-100	fs	1–100	1.1–0.8	1.5–1.3	8.2–8.5	0.3

For all compounds, Table 1 summarizes the concentration ranges probed for each substance, the measured ranges of peak maxima eKE_max_ in the eKE distributions, the full widths at half maximum (FWHMs) of the Gaussian fits, the corresponding apparent two-photon VBEs, and the concentration-induced VBE shifts ΔVBE, where applicable. The concentration-dependent VBE shifts are discussed in detail in Section 3.7.

### 3.2. Hydration effects on electron binding energies

To evaluate the effect of a droplet-phase environment on the observed VBEs, we compared our results to available gas-phase photoelectron spectra. For this comparison, only droplet spectra recorded at the lowest concentrations were considered, where contributions from short-range solute–solute interactions can be excluded.

The transition from gas-phase to aqueous-droplet environment dramatically affects the VDEs of anionic species. Phenolate exhibits a droplet-phase VDE of 8.5 eV (Fig. 4 and Table 1), representing a 6 eV increase from its reported gas-phase value of 2.3 eV.^66,67^ The hydration-induced VDE increase is due to substantial stabilization of the anionic singlet ground state through hydrogen bonding interactions with the first solvation shell of the polar protic solvent molecules, augmented by long-range electrostatic interactions with the dielectric continuum.^67^ To the best of our knowledge, no literature gas-phase references are available for catecholate and resorcinolate.

Literature references are available for the neutral species studied here, except for Triton X-100. To our knowledge, this work provides the first measurement of the aqueous-phase photoelectron spectrum and ionization energy of Triton X-100. For neutrals, a trend opposite to that of anions is expected: a decrease in VIE upon hydration as the nascent radical cation is stabilized by the solvent acting as a dielectric continuum. The magnitude of the gas-to-solution shift in VIE of neutrals – on the order of 0.5 to 1 eV – is typically much smaller than the gas-to-solution VDE shift of anions.^68,69^ The smaller magnitude is because, contrary to anions, the considerable stabilization of the nascent radical cation due to solvent rearrangement is not accounted for in the vertical transition energy.^69^

The lower magnitude of the gas-to-solution shift makes the comparison between gas-phase and aqueous-phase VIE more intricate for neutrals. Comparability of the apparent VIEs from two-photon ionization with a resonant intermediate state, calculated as VIE_meas_ = 2*hν* − eKE_max_, with gas-phase VIEs from single-photon ionization is limited. Populating the intermediate state with the resonant pump photon prior to ionization can alter overlap with the doublet ionized states, *i.e.*, transition propensities, thus affecting the measured two-photon VIEs and complicating direct comparison to the true VIE.^67^ While the apparent droplet-phase VIE of nitrobenzene of 8.4 eV is more than 1 eV lower than its corresponding gas-phase reference of 9.9 eV,^70–73^ the decrease is only minor (≤0.2 eV) for most substances. Hydroquinone and *p*-nitrophenol even exhibit a droplet-phase VIE up to 0.4 eV higher than their corresponding gas-phase values of 8.44^73^ and 8.36 eV.^71^

A more detailed understanding of the resonant 1 + 1 electron detachment/ionization processes and the resulting VBE shifts would require high-level quantum chemistry calculations, which are beyond the scope of this work.

### 3.3. Comparing electron binding energies in droplets and liquid jets

We also compared our droplet-phase VBEs with literature LJ-PES data, which are available solely for phenol, phenolate, aniline, and *p*-nitrophenol. We do note, though, that this comparison cannot necessarily be interpreted as a comparison of surface *vs.* bulk electronic structure. LJ-PES may also be considered surface-sensitive, due to effective probing depths similar to those in droplet PES (limited by (i) light penetration depth, (ii) scattering-cross-sections/escape depth, and (iii) radial solute-concentration distributions), albeit with a lower surface-to-volume ratio and possibly less equilibrated solute surface partitioning than in droplets. For phenol, the only directly comparable LJ reference – recorded *via* fs 1 + 1 two-photon ionization at 266 nm, *i.e.*, using the same ionization scheme as in our droplet experiments – yields a VBE of 8.5 ± 0.1 eV,^36^ which matches our droplet value of 8.4–8.6 eV (Table 1). For phenolate fs 1 + 1 two-photon ionization at 266 nm, a LJ VBE of 8.3 eV was reported,^74^ close to our droplet value of 8.5 eV. This agreement shows that when the same photon energy and ionization scheme is used, droplets and LJs can yield consistent results. However, the majority of available LJ reference values were recorded using single-photon ionization at much higher photon energies than employed here, yielding systematically lower VBEs (0.6–1.2 eV) than our resonance-enhanced two-photon ionization values for the reasons discussed above (Section 3.2): phenol (7.8–7.9 eV (ref. 31 and 42) *vs.* ∼8.5 eV), aniline (7.4–7.7 eV (ref. 42, 64 and 68) *vs.* 8.5 eV), and *p*-nitrophenol (7.8 eV (ref. 42) *vs.* 8.8 eV).

### 3.4. Comparison of fs and ns spectra of neutrals

Aiming to gain some insight into the photophysical behavior of the studied compounds, we compared the spectra obtained with fs and ns laser pulses for each neutral species. Both fs and ns spectra are obtained from two-photon single-pulse ionization within a single laser pulse, in which a first photon populates an electronically excited intermediate state before a second photon ionizes the system. Photoionization with the 8-ns pulse leaves substantially more time for any type of photodynamics, *e.g.*, vibrational relaxation, internal conversion (IC), inter-system crossing (ISC), and photodissociation, to occur within the duration of the laser pulse, *i.e.*, after pumping the intermediate excited singlet state with the first photon and before ionizing with the second.

For fs pulses, the two absorption events occur within the pulse duration of ≤50 fs (‘back-to-back’), whereas for ns pulses the effective delay between the first and second photon can span picoseconds to nanoseconds. Consequently, the second photon will probe a somewhat evolved intermediate state population. The ns spectra can thus be influenced not only by relaxation on the intermediate-state potential energy surface (affecting the measured two-photon VIE), but also by different electronic states populated *via* IC or ISC or by long-lived photoproducts (*e.g.*, hydrated electrons) formed after the initial excitation. This principle is routinely exploited in single-pulse ns two-photon experiments on aqueous solutions (*e.g.*, ref. 75).

In case of mere vibrational relaxation of the intermediate state within the 8-ns pulse duration, we expect to see an increase in VIE in the ns spectra. An increased apparent VIE in the ns spectrum is displayed by *p*-nitrophenol, where the photoexcitation at 267 nm is several hundred meV above the absorption maximum (Fig. 1).

At low concentrations, the ns spectra of phenol, phenolate, *p*-cresol, aniline, and the dihydroxybenzenes exhibit the same or a slightly lower VIE compared to their corresponding fs spectra (Table 1). Among them, aniline exhibits the largest difference between fs and ns spectra (0.2–0.3 eV). This shift could hint at potential photo-induced dynamics following 267-nm photoexcitation of aniline's S_1_ state (with π → π* character), which have sufficient time to occur within the 8-ns pulse duration and alter the measured VIE. According to the literature, the lifetime of aniline's 267-nm-excited S_1_ (^1^ππ*) state in aqueous solution is only ∼1 ns. From the S_1_ (^1^ππ*), hydrated electrons form *via* a ^1^πσ* state with charge-transfer-to-solvent (CTTS) character.^64,76–80^ These photochemically generated electrons fully equilibrate within tens of picoseconds,^81–83^ and the relaxed hydrated electrons may be ionized by the probe pulse. Their VBE of approximately 3.7 eV (ref. 65, 83 and 84) is in excellent agreement with the one-photon VBE of 3.7 eV, calculated as *hν* − eKE_max_, observed in the ns aniline spectra.

Since hydrated electrons are photodetached by absorption of a single photon, their binding energy is given by 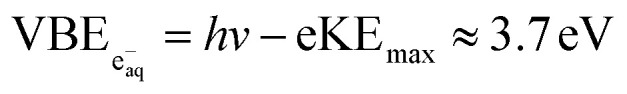 (corresponding to an eKE_max_ of 0.9 eV at 267 nm).^65,75,83,84^ When displayed on the two-photon binding-energy axis, calculated as eBE = 2*hν* − eKE (eqn (1)), the same hydrated-electron signal appears at 
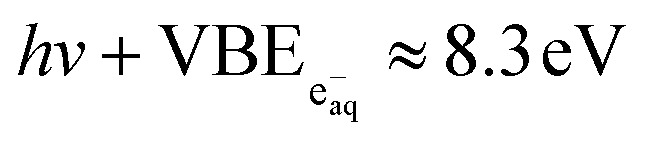
 and can therefore overlap with the solute band and bias the apparent peak maximum in the ns spectra.

Photochemically generated hydrated electrons may form similarly after 267-nm excitation of phenol, phenolate, *p*-cresol, and the dihydroxybenzenes,^19,64,74,76,79,85–89^ all of which exhibit an eKE_max_ of 0.8–1.0 eV, corresponding to a one-photon VBE of 3.6 to 3.8 eV, matching that of e^−^_aq_.

### 3.5. Comparison of fs spectra of neutral and anionic solutes

To further understand the influence of solvation and charge on the electronic structure, we compared the fs spectra of nonionic neutral species and their corresponding anionic conjugate bases at the lowest concentrations, where solute–solute interactions are negligible. As expected, the apparent VDE for the anionic conjugate bases is close to, but slightly lower than, the VIE of the corresponding neutral. In the gas phase, neutrals have VBEs several eV higher than their anionic conjugate bases. However, solvation acts toward canceling out this difference: the aqueous environment decreases the VIE of a neutral, but increases the VDE of an anion (see Section 3.2). This effect is reflected in the droplet-phase data, where the VIE of the neutral and VDE of the anionic conjugate base are similar (Table 1).

A notable difference between the spectra of neutrals and anions is the presence of a near-zero-KE signal for the neutrals, which is absent for the anionic species (see Section 3.1). The presence of this signal may be due to a higher relative amount of gas-phase signal for solutions of neutral compounds, owing to their significantly higher vapor pressures compared to ionic solutes. This interpretation would be consistent with the absence of a near-zero-KE signal for Triton X-100, which is nonionic but has a significantly lower vapor pressure than the smaller aromatic organic compounds. Alternatively, the difference could arise from the higher surface activity of neutrals,^40^ which may affect how electrons – whether from photoionization or photochemistry – escape at the interface.^60^ The charge state of the solutes may also influence the Coulombic interaction experienced by the nascent photoelectron and its escape at the droplet surface.

**Fig. 5:**
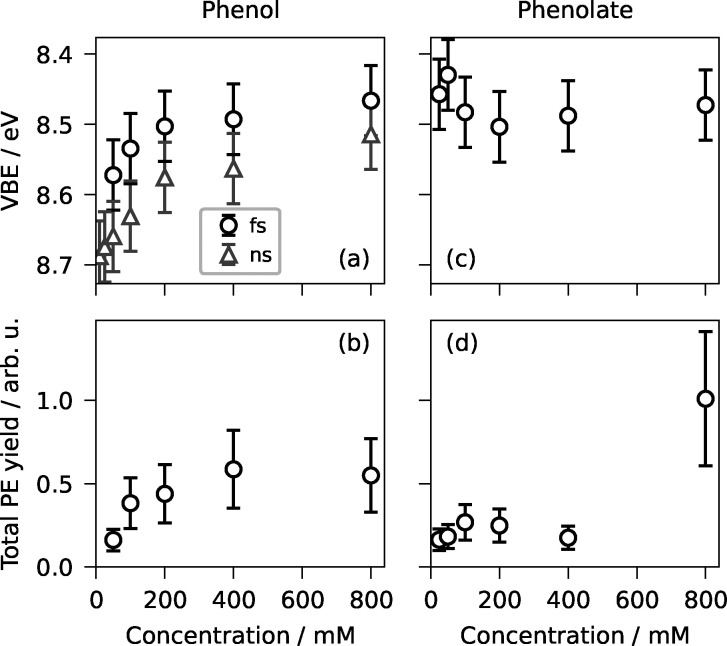
Vertical electron binding energies (top row) and total photoelectron yield (bottom row) plotted as a function of concentration for phenol (left column) and phenolate (right column). Error bars represent ±0.1 eV uncertainty in vertical binding energy from sensitivity analysis and ±40% relative uncertainty in total photoelectron yield. The uncertainty value was chosen to conservatively account for systematic uncertainties (droplet-beam density fluctuations and laser-flux determination) while remaining sufficiently small to resolve the observed, reproducible increase in signal with increasing concentration.

A (slight) systematic concentration dependence of the VBEs is observed for some neutrals but not for anions. This observation is exemplified by the phenol–phenolate comparison (Fig. 5a and c), where the neutral shows a systematic concentration-dependent shift, while the anion does not. The concentration independence may be due to Coulomb repulsion preventing ionic aromatic solutes from forming hydrogen-bonded or π-stacked dimers and larger aggregates at high concentrations that would otherwise alter the measured photoelectron spectra.^29^

### 3.6. Comparison of the VIEs of the phenolic compounds

A comparison of the VIEs among the phenolic compounds – phenol, *p*-cresol, and the dihydroxybenzenes – reveals differences in their interfacial electronic structure. In this series, hydroquinone exhibits the highest droplet-phase VIE, whereas in the gas phase, phenol, resorcinol, and catechol exhibit higher VIEs.^73^

This observation could be rationalized by considering the molecular orientation and solvation at the droplet surface. Our experiments are sensitive to the outermost molecular layer (see Section 3.8), and the interfacial hydration of the solute can significantly affect the stabilization of the cationic final state. For hydroquinone, the hydroxyl groups are located in *para* position to one another. At the surface, this means either only one hydroxyl group can be fully immersed and participate in the water hydrogen-bond network, or hydroquinone lies flat on the surface with both groups only partially hydrated, resulting in fewer hydrogen bonds.^90^ Either way, the nascent radical cation state will be subject to fewer hydrogen-bonded solvent molecules and thus be stabilized less by solute–solvent interactions than in the other phenolic compounds. This reduced stabilization may contribute to the higher VIE observed for hydroquinone in the droplet phase. A larger solvation energy of the neutral species might also be a contributing factor.

### 3.7. Concentration dependence of the VIE of neutral species

For surface-active solutes, a higher total concentration corresponds to a higher surface concentration (see also Section 3.8). Thus, the concentration dependence of the VIE for neutral species can provide insight into solute–solute interactions and self- aggregation phenomena at the droplet interface.^29^ Analysis of the apparent VIEs from fs resonance-enhanced two-photon ionization reveals that some neutral compounds exhibit a concentration-induced decrease in VBE (ΔVBE in Table 1), which is systematic but very minor relative to the experimental uncertainty of ±0.1 eV (from sensitivity analysis).

Relative to the other phenolic compounds, phenol and catechol show the largest systematic concentration-induced VIE decrease of −0.2 eV (Fig. 5), followed by resorcinol with a decrease of −0.1 eV. Hydroquinone, in contrast, shows no significant trend. Aniline also displays a similar, minor shift, which is reasonable given that its electronic structure is similar to that of phenol. Observed VIE shifts can be a result of a change in effective dielectric constant, a change in surface potential due to a net orientation of the dipoles of the surface-active molecules,^42^ or increasing solute–solute interactions and the possible formation of hydrogen-bonded or π-stacked dimers and larger aggregates with increasing concentration.^29,90–96^

Changes in surface dipole density and orientation with increasing concentration of surface-active solutes have previously been suggested to cause a slight reduction in VIE for phenol and aniline.^42^ For aromatic neutrals, the aforementioned aggregation is expected to stabilize the cationic final state through polarization and charge delocalization, thereby also slightly lowering the VIE. Both explanations are thus consistent with the modest negative ΔVBE values observed for phenol, catechol, resorcinol, and aniline in this work.

In contrast, the anionic species and other substances (except for Triton X-100) remain unchanged or show no clear trend within the uncertainty of the fit and experimental error. Triton X-100 is unique in exhibiting an inverse concentration dependence, with VIE increasing by almost 0.3 eV as concentration increases. This can be rationalized by considering the hydration environment: hydrogen-bonded water molecules that most significantly impact the stabilization of the cationic final state are located close to the phenyl ring – the chemical site at which π → π* excitation and subsequent ionization will occur – which is near the interface. At full surface coverage, the high density of long, ordered polyethylene glycol chains of the Triton X-100 molecule at the interface may inhibit solvent water molecules from binding to the phenoxy moiety due to space constraints.

### 3.8. Concentration-dependent surface coverage

Our UV PES experiments are inherently surface sensitive, probing primarily the outermost molecular layers of the droplets.^29,36–38^ For surface-active molecules, the majority of the signal originates from the outermost nanometer, as the low kinetic energy (<5 eV) photoelectrons generated have a shallow escape depth (attenuation to 1/e within <5 nm for water^65^ and <1 nm for surface-active solutes^29^). As a result, the concentration-dependent total photoelectron yield can serve as a direct measure of the fractional surface coverage of the solute *θ*,^12,26,39,40,64^ which is linked to the surface tension *γ* of a system by the semi-empirical Szyszkowski equation of state:^97–100^

2 *γ* − *γ*_water_ = *Γ*_max_ *RT *ln(1 − *θ*)

where *γ*_water_ is the surface tension of the neat solvent, *Γ*_max_ is the solute's maximum surface excess concentration (equal to the inverse of the solute's molecular footprint at the surface), *R* is the universal gas constant, and *T* the absolute temperature.^12,97,101,102^

**Fig. 6:**
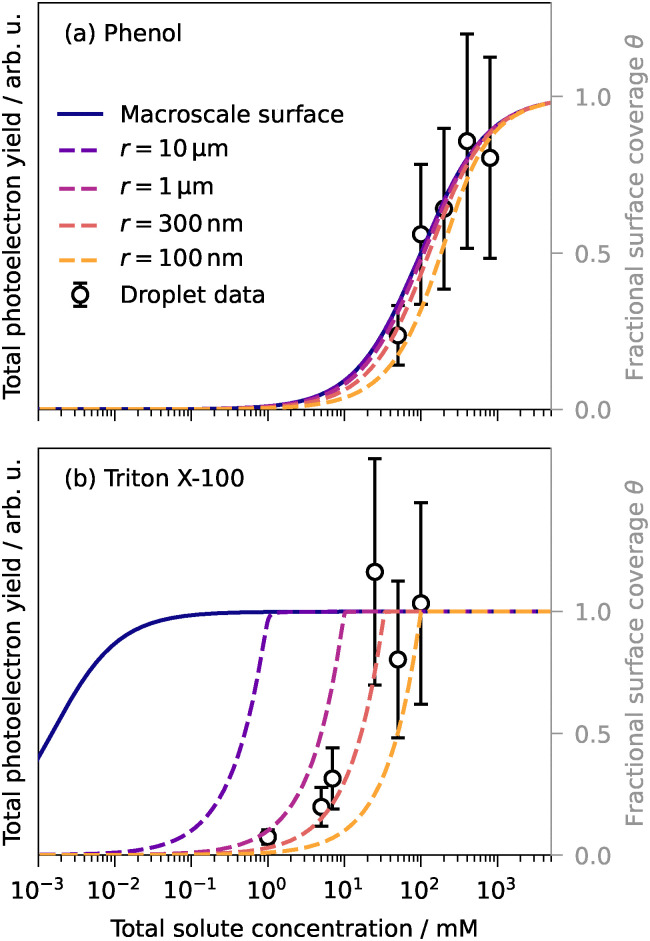
Total droplet photoelectron yield (circles) as a function of total solute concentration for aqueous phenol (top) and Triton X-100 (bottom). Error bars represent ±40% relative uncertainty in total photoelectron yield, chosen to conservatively account for systematic uncertainties (droplet-beam density fluctuations and laser-flux determination) while remaining sufficiently small to resolve the observed, reproducible increase in signal with increasing concentration. Model predictions for the concentration-dependent fractional surface coverage *θ* for a macroscale solution (Langmuir eqn (3), solid line) and submicrometer-sized droplets (finite-size model, eqn (5), dashed lines). Model predictions are based on values for *K*_ad_ and *Γ*_max_ obtained from fitting literature surface tension data^40,103^ to the Szyszkowski eqn (2): phenol:^40^*K*_ad_ = 1.0 × 10^4^ cm^3^ mol^−1^, *Γ*_max_ = 5.6 × 10^−10^ mol cm^−2^; Triton X-100:^103^*K*_ad_ = 6.6 × 10^8^ cm^3^ mol^−1^, *Γ*_max_ = 3.3 × 10^−10^ mol cm^−2^.

In accordance with recent concentration-dependent LJ and droplet XPS experiments,^26,39,40,42,64^ we expect to observe a saturation behavior of the total photoelectron yield as a function of total solute concentration, since the surface has limited capacity and will approach full coverage at sufficiently high solute concentrations. This saturation behavior was observed in the droplet PES data for phenol and Triton X-100 when plotting the total photoelectron signal as a function of solute concentration (Fig. 6). Surface saturation appears to occur at much higher concentrations of phenol than of Triton X-100. This is because phenol is only mildly surface-active, whereas Triton X-100 is a surfactant with a much higher surface activity. The difference in surface activity also manifests in the absolute photoelectron yields: although Triton X-100 has the second lowest single-photon absorption cross-section at 267 nm (Fig. 1), it exhibits the highest total photoelectron yield per unit concentration – at least an order of magnitude higher than all other substances.

For the other substances, the total photoelectron yield either scattered too much to deduce a clear trend (see, *e.g.*, phenolate in Fig. 4d) or the accessible concentration range was too narrow to make a meaningful statement. Some substances, such as resorcinol and catechol, even show a decrease in signal with increasing concentration, possibly due to increased clogging at the pressure-limiting aperture. The total photoelectron yields of resorcinolate and aniline increase monotonically with concentration; however, for aniline, only three data points were recorded, and resorcinolate lacks macroscale flat-surface tension data for reference. Thus, only data for phenol and Triton X-100 are shown.

For macroscale solutions (that are dilute and premicellar), the fractional surface coverage *θ* as a function of bulk solute concentration *c*_bulk_ is typically well described by the Langmuir adsorption isotherm:3
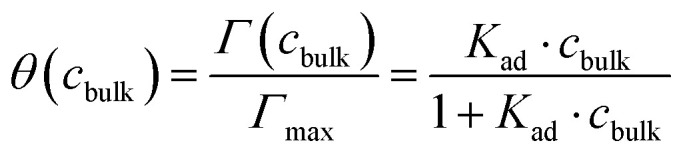
4
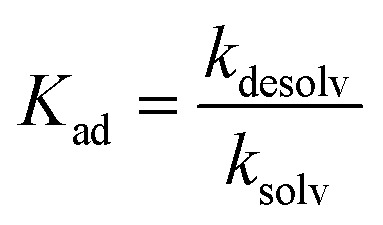
where *Γ* is the concentration-dependent equilibrium surface excess concentration of the solute and *K*_ad_ is the Langmuir adsorption equilibrium constant, expressed as the ratio of the rate coefficients for adsorption to the surface, *k*_desolv_, and desorption to the bulk, *k*_solv_.^26,102^ For phenol, the prediction of surface coverage for a macroscale system from the Langmuir equation is within the experimental error of the measured total droplet photoelectron yield (Fig. 6a), but for Triton X-100, a significant discrepancy is observed (Fig. 6b). For Triton X-100, full surface coverage – and thus minimal surface tension (eqn (2)) – occurs at much higher concentrations in droplets than in a macroscale solution. In macroscale solution, 50% surface coverage is reached at a total Triton X-100 concentration of ∼10^−3^ mM, whereas in the droplets studied here, 50% coverage is not reached before ∼10^1^ mM (Fig. 6). This demonstrates that the surfactant concentration required to reach this surface coverage and the corresponding surface tension is approximately 10^4^ times higher in 300-nm droplets than in macroscale solution.

This discrepancy arises because the bulk concentration within the droplet is substantially perturbed by the droplet interface.^26,102^ The large surface-to-volume ratio leads to solute depletion in the droplet interior, often referred to as “bulk depletion”, and a breakdown of the Langmuir equation for describing the equilibrium surface concentration/coverage. While the underlying thermodynamics of the surface partitioning equilibrium are the same in macroscale systems and submicrometer-sized droplets, the surface-to-volume ratio affects the partitioning behavior and necessitates accounting for the effect of bulk depletion.^26,102^

A model for the equilibrated surfactant coverage at the surface of finite-size droplets that accounts for interior solute depletion was derived by Wilson and Prophet^26,102^ (similar to that developed by Alvarez *et al.*^104^), showing that the surface coverage in (sub)micrometer-sized droplets is a strong function of the average droplet radius *r*:5
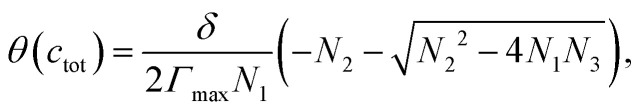
where*N*_1_ = 3*k*_desolv_*δ*^2^,*N*_2_ = −3*k*_desolv_*Γ*_max_*δ* − *k*_desolv_*c*_tot_*δr* − *k*_solv_*δr*,*N*_3_ = *k*_desolv_*Γ*_max_*c*_tot_*r*.Here, *δ* is the interface thickness (assumed to be 1 nm (ref. 102)) and *c*_tot_ is the total solute concentration. Note that *c*_tot_ = *c*_bulk_ for macroscale solutions, where the Langmuir equation holds, but not for submicrometer droplets, where bulk depletion is considerable. The model predictions for the concentration-dependent fractional surface coverage for submicrometer-sized droplets are displayed by the dashed lines for different droplet sizes in Fig. 6. Although the finite-size model (eqn (5)) is expressed in terms of the individual rate constants *k*_solv_ and *k*_desolv_, they do not independently influence the equilibrium surface coverage. The equilibrium surface coverage is solely determined by their ratio, *i.e.*, the adsorption equilibrium constant *K*_ad_ (eqn (4)), rendering the specific absolute values of the rate constants inconsequential for the calculated equilibrium curves. For the Triton X-100 data, the best match between model and experimental data is achieved for a droplet radius of 300 nm, in excellent agreement with the mean droplet size determined *in situ* from the VMI (Section 2).

The comparison of Triton X-100 and phenol reveals that the extent of surface propensity significantly influences the magnitude of the surface tension difference between macroscale solutions and submicrometer aqueous aerosol droplets. For phenol, container size has a smaller effect because of its lower surface activity compared to that of the surfactant Triton X-100. Thus, for only mildly surface-active amphiphilic benzene derivatives, the droplet PES data can currently not distinguish between the changes in surface concentration/coverage/tension for a submicrometer-sized droplet *versus* a macroscale solution. Our experimental results support previous studies in demonstrating that the surface-to-volume ratio determines the surface tension in microscopic surfactant-containing droplets.^12^

### 3.9. Comparison of electron binding energies in phenol–water clusters and droplets

The smaller the water–solute system becomes, the larger its surface-to-volume ratio, the more selectively photoelectron spectroscopy probes interfacial solute. To investigate the influence of system size and surface-to-volume ratio on the valence electronic structure of hydrated aromatic molecules, we compared the photoelectron spectra of water clusters (mean size ∼170 water molecules, ∼2 nm diameter) with those from aqueous droplets (∼300 nm diameter). Phenol was chosen as a proxy solute for this comparison.

Two-photon photoelectron spectra of phenol–water clusters were recorded, and the cluster size distribution, retrieved from time-of-flight mass spectra, revealed a mass-weighted mean cluster size of approximately 170 water molecules per phenol molecule. Assuming a spherical cluster with bulk density, this corresponds to an average diameter of about 2 nm. Given that the typical surface layer thickness is 1 nm (Section 3.8),^102^ these clusters can be considered to be composed almost entirely of surface molecules. In other words, every molecule in the cluster is at or near the surface, providing a model system for a purely interfacial environment.

A key experimental difference is that the cluster spectra were recorded at laser peak intensities (GW cm^−2^) at least an order of magnitude higher than those used for the droplet measurements. At these higher intensities, the cluster photoelectron spectra exhibit a notable laser-power dependence: as the laser power increases, the spectral maximum shifts to lower eKEs, corresponding to higher eBEs. The spectrum recorded at the lowest laser intensity of ∼5 GW cm^−2^ peaks near 0.5 eV eKE, whereas at five times higher intensity, the peak shifts to near-zero eKE. This intensity-dependent shift likely originates from a combination of physical phenomena inherent to multiphoton ionization in dense media with intense fs pulses. Plausible causes could include space-charge effects, where repulsion within the dense cloud of photoelectrons reduces their final eKE, and sequential ionization, where a given cluster becomes multiply charged during the laser pulse, increasing the Coulombic attraction that subsequent photoelectrons must overcome to escape.^105–107^ The latter is plausible since an electron escaping from a singly charged sphere of 2 nm radius would lose ∼0.7 eV of kinetic energy to overcome the Coulomb potential.

**Fig. 7:**
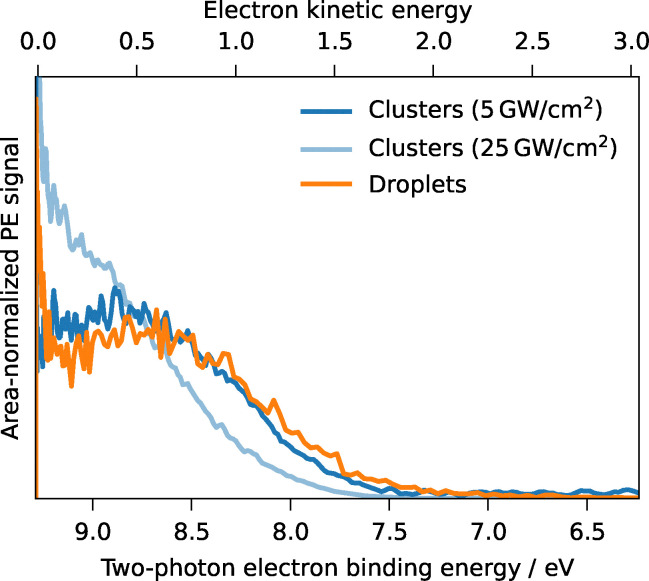
Photoelectron spectra of phenol–water clusters (blue) and phenol (50 mM) in aqueous droplets (orange) from single-pulse femtosecond 1 + 1 resonance-enhanced two-photon ionization at 267 nm.

To ensure a meaningful comparison and avoid biases introduced by these high-intensity effects, we exclusively use the cluster spectrum recorded at the lowest laser intensity to compare with the droplet spectrum. For the droplet reference, we selected the spectrum at a low phenol concentration (50 mM) in droplets, where phenol molecules are isolated at the surface, phenol–phenol interactions are minimal, and aggregation is absent.^29^ This allowed us to test whether the photoelectron spectra of phenol in clusters and droplets are similar under conditions where both systems probe primarily surface phenol. As shown in Fig. 7, this low-intensity cluster spectrum and the low-concentration droplet spectrum overlap remarkably well, with nearly identical peak positions and widths within experimental uncertainties. The only notable difference is the pronounced peak at near-zero eKE in the droplet spectrum, which is absent in the low-intensity cluster spectrum (see Section 3.1). This striking similarity demonstrates that the VIE of phenol at the droplet–vacuum interface is not significantly different from its VIE in small clusters of water, reinforcing the idea that our droplet measurements indeed probe molecules in a surface environment. Moreover, this result underlines that there is practically no spectral contribution from phenol aggregates or phenol–phenol interactions in the droplet spectra at the lowest phenol concentration.

The reported static two-photon photoelectron spectra lay the groundwork for future studies that aim to probe and compare the photodynamics in clusters and droplets using time-resolved photoelectron spectroscopy. By showing that the electronic structure of interfacial aromatic solutes converges for gas-phase clusters and dilute droplets, this work provides a crucial bridge between two key experimental paradigms and validates the use of computationally tractable cluster models to understand complex interfacial phenomena.

## 4. Conclusion

This study systematically investigated valence photoionization of 13 mono- or di-substituted benzenes at the interface of aqueous submicron aerosol droplets. UV droplet photoelectron imaging enabled solute-selective and interface-sensitive probing, accessing the lowest electron binding energies of interfacial aqueous aromatic solutes. The quantitative experimental data presented here provide essential benchmarks for computational chemistry models of interfacial electronic structure in droplets and pave the way for a better understanding of the connection between altered electronic ground and excited-state stabilities at the molecular scale and observed photochemical reaction rate acceleration at the macroscale.

Solvation-induced VBE shifts of anions and neutrals follow predictable patterns based on stabilizing interactions of ionic initial or final states with the polar protic solvent. The droplet environment increases the VDE of anionic species by ∼6 eV, while decreasing the VIE of neutral species by 0.5–1 eV relative to the corresponding gas-phase values. Concentration-dependent surface accumulation and resulting solute–solute interactions may modify the apparent VIEs of neutral aromatic solutes, while ionic aromatic solutes show no concentration-induced VDE shift, suggesting they remain sufficiently separated even at concentrations exceeding 1 M due to electrostatic repulsion.

The interfacial electronic structure converges between clusters and droplets at low surface coverage for the phenol–water system. Phenol–water clusters (∼170 water molecules) and dilute droplets (50 mM) exhibit eBE spectra identical within experimental uncertainties, confirming surface selectivity in UV droplet PES of substituted benzenes and validating cluster studies as models for interfacial solvation.

The large surface-to-volume ratio controls surfactant partitioning behavior in submicrometer-sized droplets. Achieving a given surface tension (corresponding to 50% surface coverage) in *r* = 300 nm droplets requires 10^4^-fold higher Triton X-100 concentrations (∼10^1^ mM) compared to macroscopic solutions (∼10^−3^ mM), quantitatively explained by finite-size models that account for bulk depletion (eqn (5)).

Comparison of femtosecond and nanosecond single-pulse two-photon spectra hints at compound-specific photophysics following 267-nm photoexcitation, such as CTTS-mediated electron ejection generating hydrated electrons (VBE ≈ 3.7 eV) for aqueous aniline within the experimental 8 ns time scale. This lays the foundation for future time-resolved droplet photoelectron imaging studies, which can bring a fresh perspective to research questions such as the debated 10 000-fold rate acceleration of phenol's 267-nm photodissociation at the water interface.^18,19^

## Conflicts of interest

There are no conflicts to declare.

## Data Availability

Data for this article are available at the ETH Research Collection at https://doi.org/10.3929/ethz-c-000787110.^108^

## References

[cit1] Lewis A. C., Carslaw N., Marriott P. J., Kinghorn R. M., Morrison P., Lee A. L., Bartle K. D., Pilling M. J. (2000). Nature.

[cit2] Cabrera-Perez D., Taraborrelli D., Sander R., Pozzer A. (2016). Atmos. Chem. Phys..

[cit3] Taraborrelli D., Cabrera-Perez D., Bacer S., Gromov S., Lelieveld J., Sander R., Pozzer A. (2021). Atmos. Chem. Phys..

[cit4] Lau N. A., Ghosh D., Bourne-Worster S., Kumar R., Whitaker W. A., Heitland J., Davies J. A., Karras G., Clark I. P., Greetham G. M., Worth G. A., Orr-Ewing A. J., Fielding H. H. (2024). J. Am. Chem. Soc..

[cit5] Desyaterik Y., Sun Y., Shen X., Lee T., Wang X., Wang T., Collett Jr. J. L. (2013). J. Geophys. Res.: Atmos..

[cit6] Laskin A., Laskin J., Nizkorodov S. A. (2015). Chem. Rev..

[cit7] Reed Harris A. E., Pajunoja A., Cazaunau M., Gratien A., Pangui E., Monod A., Griffith E. C., Virtanen A., Doussin J.-F., Vaida V. (2017). J. Phys. Chem. A.

[cit8] Fleming L. T., Lin P., Roberts J. M., Selimovic V., Yokelson R., Laskin J., Laskin A., Nizkorodov S. A. (2020). Atmos. Chem. Phys..

[cit9] Andreae M. O., Gelencsér A. (2006). Atmos. Chem. Phys..

[cit10] Formenti P., Elbert W., Maenhaut W., Haywood J., Osborne S., Andreae M. O. (2003). J. Geophys. Res.: Atmos..

[cit11] Corral Arroyo P., David G., Alpert P. A., Parmentier E. A., Ammann M., Signorell R. (2022). Science.

[cit12] Bain A., Ghosh K., Prisle N. L., Bzdek B. R. (2023). ACS Cent. Sci..

[cit13] Bain R. M., Pulliam C. J., Thery F., Cooks R. G. (2016). Angew. Chem., Int. Ed..

[cit14] Wei Z., Li Y., Cooks R. G., Yan X. (2020). Annu. Rev. Phys. Chem..

[cit15] Li K., Gong K., Liu J., Ohnoutek L., Ao J., Liu Y., Chen X., Xu G., Ruan X., Cheng H., Han J., Sui G., Ji M., Valev V. K., Zhang L. (2022). Cell Rep. Phys. Sci..

[cit16] Marsh B. M., Iyer K., Cooks R. G. (2019). J. Am. Soc. Mass Spectrom..

[cit17] Wilson K. R., Prophet A. M., Rovelli G., Willis M. D., Rapf R. J., Jacobs M. I. (2020). Chem. Sci..

[cit18] Kusaka R., Nihonyanagi S., Tahara T. (2021). Nat. Chem..

[cit19] Jordan C. J. C., Lowe E. A., Verlet J. R. R. (2022). J. Am. Chem. Soc..

[cit20] George C., Ammann M., D'Anna B., Donaldson D. J., Nizkorodov S. A. (2015). Chem. Rev..

[cit21] Anglada J. M., Martins-Costa M. T., Francisco J. S., Ruiz-López M. F. (2020). J. Am. Chem. Soc..

[cit22] Ge Q., Liu Y., Li K., Xie L., Ruan X., Wang W., Wang L., Wang T., You W., Zhang L. (2023). Angew. Chem., Int. Ed..

[cit23] Dai Q., Zhang X., Lin J., Cui T., Wang W., Yu G., Cao H., Zhao H. (2024). Green Chem..

[cit24] Mohajer M. A., Basuri P., Evdokimov A., David G., Zindel D., Miliordos E., Signorell R. (2025). Science.

[cit25] Kappes K. J., Deal A. M., Jespersen M. F., Blair S. L., Doussin J.-F., Cazaunau M., Pangui E., Hopper B. N., Johnson M. S., Vaida V. (2021). J. Phys. Chem. A.

[cit26] Deal A. M., Prophet A. M., Warkander S., Foreman M. M., Kim P., Neumark D. M., Ahmed M., Wilson K. R. (2025). Phys. Chem. Chem. Phys..

[cit27] Frandsen B. N., Vaida V. (2022). J. Phys. Chem. A.

[cit28] Anglada J. M., Martins-Costa M., Ruiz-López M. F., Francisco J. S. (2014). Proc. Natl. Acad. Sci. U. S. A..

[cit29] Heitland J., Lee J. C., Ban L., Abma G. L., Fortune W. G., Fielding H. H., Yoder B. L., Signorell R. (2024). J. Phys. Chem. A.

[cit30] Petersen-Sonn E. A., Jespersen M. F., Johnson M. S., Mikkelsen K. V. (2025). ACS Earth Space Chem..

[cit31] Ghosh D., Roy A., Seidel R., Winter B., Bradforth S., Krylov A. I. (2012). J. Phys. Chem. B.

[cit32] Seidel R., Winter B., Bradforth S. E. (2016). Annu. Rev. Phys. Chem..

[cit33] Roy A., Seidel R., Kumar G., Bradforth S. E. (2018). J. Phys. Chem. B.

[cit34] Kumar G., Roy A., McMullen R. S., Kutagulla S., Bradforth S. E. (2018). Faraday Discuss..

[cit35] Signorell R., Winter B. (2022). Phys. Chem. Chem. Phys..

[cit36] Scholz M. S., Fortune W. G., Tau O., Fielding H. H. (2022). J. Phys. Chem. Lett..

[cit37] Tau O., Henley A., Boichenko A. N., Kleshchina N. N., Riley R., Wang B., Winning D., Lewin R., Parkin I. P., Ward J. M., Hailes H. C., Bochenkova A. V., Fielding H. H. (2022). Nat. Commun..

[cit38] Fortune W. G., Scholz M. S., Fielding H. H. (2022). Acc. Chem. Res..

[cit39] Prisle N. L. (2024). Acc. Chem. Res..

[cit40] Richter C., Dupuy R., Trinter F., Buttersack T., Cablitz L., Gholami S., Stemer D., Nicolas C., Seidel R., Winter B., Bluhm H. (2024). Phys. Chem. Chem. Phys..

[cit41] Buttersack T., Gladich I., Gholami S., Richter C., Dupuy R., Nicolas C., Trinter F., Trunschke A., Delgado D., Corral Arroyo P., Parmentier E. A., Winter B., Iezzi L., Roose A., Boucly A., Artiglia L., Ammann M., Signorell R., Bluhm H. (2024). Nat. Commun..

[cit42] Yamamoto Y. I., Hirano T., Ishiyama T., Morita A., Suzuki T. (2025). J. Am. Chem. Soc..

[cit43] Yoder B. L., West A. H. C., Schläppi B., Chasovskikh E., Signorell R. (2013). J. Chem. Phys..

[cit44] Signorell R., Goldmann M., Yoder B. L., Bodi A., Chasovskikh E., Lang L., Luckhaus D. (2016). Chem. Phys. Lett..

[cit45] Lin P. C., Wu Z. H., Chen M. S., Li Y. L., Chen W. R., Huang T. P., Lee Y. Y., Wang C. C. (2017). J. Phys. Chem. B.

[cit46] Ban L., Yoder B. L., Signorell R. (2020). Annu. Rev. Phys. Chem..

[cit47] Ban L., Tang H., Yoder B. L., Signorell R. (2022). Faraday Discuss..

[cit48] Kurahashi N., Karashima S., Tang Y., Horio T., Abulimiti B., Suzuki Y.-I., Ogi Y., Oura M., Suzuki T. (2014). J. Chem. Phys..

[cit49] Riley J. W., Wang B., Parkes M. A., Fielding H. H. (2019). Rev. Sci. Instrum..

[cit50] Niesner R., Heintz A. (2000). J. Chem. Eng. Data.

[cit51] Plugatyr A., Svishchev I. M. (2011). J. Phys. Chem. B.

[cit52] Faubel M., Steiner B., Toennies J. P. (1997). J. Chem. Phys..

[cit53] Winter B., Weber R., Widdra W., Dittmar M., Faubel M., Hertel I. V. (2004). J. Phys. Chem. A.

[cit54] Gartmann T. E., Hartweg S., Ban L., Chasovskikh E., Yoder B. L., Signorell R. (2018). Phys. Chem. Chem. Phys..

[cit55] Eppink A. T. J. B., Parker D. H. (1997). Rev. Sci. Instrum..

[cit56] Dasch C. J. (1992). Appl. Opt..

[cit57] Gibson S. , Hickstein D. D., Yurchak R., Ryazanov M., Das D. and Shih G., PyAbel/PyAbel: V0.9.0, Zenodo, 2022

[cit58] Smith J. D., Cappa C. D., Drisdell W. S., Cohen R. C., Saykally R. J. (2006). J. Am. Chem. Soc..

[cit59] Goy C., Potenza M. A. C., Dedera S., Tomut M., Guillerm E., Kalinin A., Voss K. O., Schottelius A., Petridis N., Prosvetov A., Tejeda G., Fernández J. M., Trautmann C., Caupin F., Glasmacher U., Grisenti R. E. (2018). Phys. Rev. Lett..

[cit60] Ban L., Gartmann T. E., Yoder B. L., Signorell R. (2020). Phys. Rev. Lett..

[cit61] Even U. (2015). EPJ Technol. Instrum..

[cit62] Horn K., Tsizin S., Ban L., Chasovskikh E., Yoder B. L., Calegari F., Signorell R. (2025). Phys. Rev. Res..

[cit63] Ban L., Tang H., Heitland J., West C. W., Yoder B. L., Thanopulos I., Signorell R. (2024). Nanoscale.

[cit64] West C. W., Nishitani J., Higashimura C., Suzuki T. (2021). Mol. Phys..

[cit65] Luckhaus D., Yamamoto Y., Suzuki T., Signorell R. (2017). Sci. Adv..

[cit66] Woodhouse J. L., Henley A., Parkes M. A., Fielding H. H. (2019). J. Phys. Chem. A.

[cit67] Boichenko A. N., Bochenkova A. V. (2023). J. Chem. Theory Comput..

[cit68] Tentscher P. R., Seidel R., Winter B., Guerard J. J., Arey J. S. (2015). J. Phys. Chem. B.

[cit69] Schewe H. C., Brezina K., Kostal V., Mason P. E., Buttersack T., Stemer D. M., Seidel R., Quevedo W., Trinter F., Winter B., Jungwirth P. (2022). J. Phys. Chem. B.

[cit70] Kobayashi T., Nagakura S. (1974). Bull. Chem. Soc. Jpn..

[cit71] Kobayashi T., Nagakura S. (1975). J. Electron Spectrosc. Relat. Phenom..

[cit72] Rabalais J. W. (1972). J. Chem. Phys..

[cit73] Palmer M. H., Moyes W., Speirs M., Ridyard J. N. A. (1979). J. Mol. Struct..

[cit74] Robertson K., Fortune W. G., Davies J. A., Boichenko A. N., Scholz M. S., Tau O., Bochenkova A. V., Fielding H. H. (2023). Chem. Sci..

[cit75] Shreve A. T., Yen T. A., Neumark D. M. (2010). Chem. Phys. Lett..

[cit76] Sobolewski A. L., Domcke W. (2001). J. Phys. Chem. A.

[cit77] Sobolewski A. L., Domcke W., Dedonder-Lardeux C., Jouvet C. (2002). Phys. Chem. Chem. Phys..

[cit78] Ashfold M. N. R., Cronin B., Devine A. L., Dixon R. N., Nix M. G. D. (2006). Science.

[cit79] Ashfold M. N., King G. A., Murdock D., Nix M. G., Oliver T. A., Sage A. G. (2010). Phys. Chem. Chem. Phys..

[cit80] Lamas I., González J., Longarte A., Montero R. (2023). J. Chem. Phys..

[cit81] Kloepfer J. A., Vilchiz V. H., Lenchenkov V. A., Germaine A. C., Bradforth S. E. (2000). J. Chem. Phys..

[cit82] Gartmann T. E., Ban L., Yoder B. L., Hartweg S., Chasovskikh E., Signorell R. (2019). J. Phys. Chem. Lett..

[cit83] Ban L., West C. W., Chasovskikh E., Gartmann T. E., Yoder B. L., Signorell R. (2020). J. Phys. Chem. A.

[cit84] Nishitani J., Ichi Yamamoto Y., West C. W., Karashima S., Suzuki T. (2019). Sci. Adv..

[cit85] Feitelson J., Hayon E., Treinin A. (1973). J. Am. Chem. Soc..

[cit86] Oliver T. A., Zhang Y., Roy A., Ashfold M. N., Bradforth S. E. (2015). J. Phys. Chem. Lett..

[cit87] Riley J. W., Wang B., Woodhouse J. L., Assmann M., Worth G. A., Fielding H. H. (2018). J. Phys. Chem. Lett..

[cit88] Henley A., Riley J. W., Wang B., Fielding H. H. (2019). Faraday Discuss..

[cit89] Jordan C. J. C., Coons M. P., Herbert J. M., Verlet J. R. R. (2024). Nat. Commun..

[cit90] Yang H., Gladich I., Boucly A., Artiglia L., Ammann M. (2022). Environ. Sci.: Atmos..

[cit91] Lehrer S. S., Fasman G. D. (1965). J. Am. Chem. Soc..

[cit92] Smith V. R., Samoylova E., Ritze H. H., Radloff W., Schultz T. (2010). Phys. Chem. Chem. Phys..

[cit93] Zhang Y., Oliver T. A. A., Ashfold M. N. R., Bradforth S. E. (2012). Faraday Discuss..

[cit94] Malongwe J. K., Nachtigallová D., Corrochano P., Klán P. (2016). Langmuir.

[cit95] Grieco C., Kohl F. R., Zhang Y., Natarajan S., Blancafort L., Kohler B. (2019). Photochem. Photobiol..

[cit96] Turner M. A. P., Turner R. J., Horbury M. D., Hine N. D. M., Stavros V. G. (2019). J. Chem. Phys..

[cit97] von Szyszkowski B. (1908). Z. Phys. Chem..

[cit98] Langmuir I. (1917). J. Am. Chem. Soc..

[cit99] Chang C.-H., Franses E. I. (1995). Colloids Surf., A.

[cit100] Menger F. M., Rizvi S. A. A. (2011). Langmuir.

[cit101] Meissner H. P., Michaels A. S. (1949). Ind. Eng. Chem..

[cit102] Wilson K. R., Prophet A. M. (2024). Annu. Rev. Phys. Chem..

[cit103] Bzdek B. R., Reid J. P., Malila J., Prisle N. L. (2020). Proc. Natl. Acad. Sci. U. S. A..

[cit104] Alvarez N. J., Walker L. M., Anna S. L. (2012). Soft Matter.

[cit105] Powell J. A., Summers A. M., Liu Q., Robatjazi S. J., Rupp P., Stierle J., Trallero-Herrero C., Kling M. F., Rudenko A. (2019). Opt. Express.

[cit106] Petrov G. M., Davis J., Velikovich A. L., Kepple P., Dasgupta A., Clark R. W. (2005). Phys. Plasmas.

[cit107] Arbeiter M., Fennel T. (2011). New J. Phys..

[cit108] Heitland J. , Lee J. C., Abma G. L., Schulke S., Friz J.-H., Tsizin S., Yoder B. L. and Signorell R., Data Collection: Photoelectron Imaging of Substituted Benzenes in Aqueous Aerosol Droplets, ETH Research Collection, https://doi.org/10.3929/ethz-c-000787110, 2026 10.1039/d5cp04376j PMC12911998 41701145

